# *Populus euphratica* Apyrases Increase Drought Tolerance by Modulating Stomatal Aperture in Arabidopsis

**DOI:** 10.3390/ijms22189892

**Published:** 2021-09-13

**Authors:** Yanli Zhang, Yuanling Sun, Xiaojing Liu, Jiayin Deng, Jun Yao, Yinan Zhang, Shurong Deng, Huilong Zhang, Nan Zhao, Jinke Li, Xiaoyang Zhou, Rui Zhao, Shaoliang Chen

**Affiliations:** 1 Beijing Advanced Innovation Center for Tree Breeding by Molecular Design, College of Biological Sciences and Technology, Beijing Forestry University, Beijing 100083, China; zhangyl@bjfu.edu.cn (Y.Z.); 18003251998@163.com (Y.S.); lxj552541@bjfu.edu.cn (X.L.); dengjiayin12345@163.com (J.D.); yaojun990@126.com (J.Y.); zhaonan19880921@126.com (N.Z.); jinkeli@bjfu.edu.cn (J.L.); zhouxiaoyang@bjfu.edu.cn (X.Z.); ruizhao926@126.com (R.Z.); 2 Forestry Institute of New Technology, Chinese Academy of Forestry, Beijing 100091, China; xhzyn007@163.com; 3 State Key Laboratory of Tree Genetics and Breeding, The Research Institute of Forestry, Chinese Academy of Forestry, Beijing 100091, China; danceon@126.com; 4 Research Center of Saline and Alkali Land of National Forestry and Grassland Administration, Chinese Academy of Forestry, Beijing 100091, China; hlzhang2018@126.com

**Keywords:** poplar, extracellular ATP, apyrase 1, apyrase 2, abscisic acid, stomatal aperture, water stress, water loss, light, guard cell

## Abstract

Stomatal regulation is crucial to reduce water consumption under drought conditions. Extracellular ATP (eATP) serves as a signaling agent in stomatal regulation; however, it is less known whether the eATP mediation of stomatal aperture is linked to apyrases (APYs), the principal enzymes that control the concentration of eATP. To clarify the role of APYs in stomatal control, *PeAPY1* and *PeAPY2* were isolated from *Populus euphratica* and transferred into Arabidopsis. Compared with the wild-type Arabidopsis and loss-of-function mutants (*Atapy1* and *Atapy2*), *PeAPY1*- and *PeAPY2*-transgenic plants decreased stomatal aperture under mannitol treatment (200 mM, 2 h) and reduced water loss during air exposure (90 min). The role of apyrase in stomatal regulation resulted from its control in eATP-regulated stomatal movements and increased stomatal sensitivity to ABA. The bi-phasic dose-responses to applied nucleotides, i.e., the low ATP (0.3–1.0 mM)-promoted opening and high ATP (>2.0 mM)-promoted closure, were both restricted by *P. euphratica* apyrases. It is noteworthy that eATP at a low concentration (0.3 mM) counteracted ABA action in the regulation of stomatal aperture, while overexpression of *PeAPY1* or *PeAPY2* effectively diminished eATP promotion in opening, and consequently enhanced ABA action in closure. We postulate a speculative model of apyrase signaling in eATP- and ABA-regulated stomatal movements under drought.

## 1. Introduction

Under global climate change, drought is becoming more and more frequent and longer lasting worldwide [1,2,3,4]. Genetic engineering is an effective strategy for increasing drought resistance in herbaceous and woody species [3,4]. The conserved signaling pathway attributed to stomatal movement can be a suitable target for genetic engineering since stomatal regulation is crucial to reduce water consumption when root water uptake is restrained [4,5,6,7,8,9]. Extracellular ATP (eATP) has been widely considered as a signaling agent in plant growth, development and stress response [10,11,12,13,14,15,16,17,18]. Accumulating evidence has shown that eATP regulates stomatal aperture in *Arabidopsis thaliana* and *Vicia faba* [19,20,21,22]. Therefore, it can be inferred that eATP has a potential role in mediating water status through regulation of stomatal aperture under drought conditions. Exogenously applied ATP in hydrolyzable and nonhydrolyzable forms regulates stomatal aperture in a bi-phasic pattern [19]. Application of ATPγS at a low dose, 5 or 15 μM, in the dark induces stomatal opening, while application of 25 µM ATPγS or more does not have an effect in the dark [19]. Hao et al. and Wang et al. observed ATP-promoted stomatal opening in light when treating leaves with ATP at concentrations of 0.1, 0.3, 0.5 and 1.0 mM [20,22], while application of ATPγS of more than 150 μM, e.g., 200 or 250 μM [19], or ATP > 1.5 mM [21], induces stomatal closure in the light. eATP is shown to induce the production of second messengers, which are involved in the stomatal control, such as reactive oxygen species (ROS), cytosolic Ca^2+^ ([Ca^2+^]_cyt_) and NO [19,20,21,22]. Hydrogen peroxide (H_2_O_2_) and NO mediate stomatal closure induced by 200 μM ATPγS [19]. eATP-promoted stomatal opening is also mediated by ROS, [Ca^2+^]_cyt_, heterotrimeric G protein α subunit (Gα) and plasma membrane (PM) H^+^-ATPase [20]. Wang et al. suggested that eATP may promote stomatal opening via ROS that regulate guard cell plasma membrane (PM) Ca^2+^ channels [22]. Recently, Chen et al. showed that eATP elicits DORN1-mediated RBOHD phosphorylation to regulate stomatal aperture [23].

eATP mediation of stomatal aperture is linked to the extracellular apyrases (or ectoapyrases, APYs), the principal enzymes that limit the ATP accumulation in the extracellular matrix (ECM) [24,25,26]. APYs play important roles in the signaling steps in stomatal movements. Application of soluble potato apyrase, which would decrease endogenous eATP levels, blocked stomatal opening of *A. thaliana* in the light [20]. The finding is in agreement with Clark et al., who have shown that moderate inhibition of ectoapyrase activity by application of low concentrations of chemical apyrase inhibitors, such as NGXT 191 (1.5 μg/mL) and apyrase inhibitor #13 (1.5 μg/mL), resulted in stomatal opening in darkness [21]. However, the role of apyrase in stomatal control is less known under water stress conditions.

Abscisic acid (ABA) induced by water deficit triggers a signaling cascade, leading to stomatal closure [27]. ABA binds to the pyrabactin resistance 1 (PYR1)/PYR1-like (PYL)/regulatory components of ABA receptors (RCAR) to form a complex with PP2Cs (Type 2C phosphatases). This subsequently results in phosphorylation and activation of SnRK2s (kinase), which activates down-stream transcription factors targeting stomatal control and stress acclimation [28,29,30]. It is also shown that eATP and apyrase interact with ABA in stomatal regulation. Clark et al. found that combining low levels of ABA with low levels of ATPγS resulted in stomatal closure, and the added apyrase could block ABA-induced stomatal closure in light [21]. This suggests that removing the eATP by apyrase would affect the ABA-induced closure. The result is in accordance with the finding that treatment of epidermal peels with apyrase inhibitor NGXT 191 (7.5 μg/mL), which increased eATP levels, induced stomatal closure [19]. However, RNAi suppression of apyrase expression leads to increased stomatal aperture in response to light [19], which is in contrast to the results showing that chemical inhibition of apyrase promotes stomatal closure [19]. It is necessary to clarify the role of apyrase in eATP- and ABA-mediated stomatal aperture with genetically modified plants differing in apyrase expression.

*Populus euphratica* is a tree species that can adapt to harsh temperature conditions in saline and alkaline desert sites. Moderate water stress increased concentrations of soluble carbohydrates and polyols, which benefits *P. euphratica* leaves to maintain cell turgor through increased osmotic pressure [31]. Drought-increased ABA also contributes to stomatal control and drought tolerance in poplars [4,32]. In the present study, we attempted to evaluate the signaling of apyrase in stomatal movements regulated by eATP and the drought stress signal, ABA. *PeAPY1* and *PeAPY2* genes have been cloned from *P. euphratica* and transferred into a model species, Arabidopsis and tobacco [26,33,34,35]. We have previously shown that the overexpression of *P. euphratica* apyrase genes increased cold, salt and drought tolerance in transgenic plants [26,33,34,35]. Similarly, ectopic expression of a pea apyrase enhances root system architecture and drought survival in Arabidopsis and soybean [36]. In this study, we used transgenic lines of Arabidopsis (PeAPY1-OE and PeAPY2-OE) to testify to the role of *P. euphratica* apyrase in stomatal control. The Arabidopsis loss-of-function mutants for *APY1* and *APY2* (*Atapy1* and *Atapy2*) were used as negative controls. Our data showed that *PeAPY1*- and *PeAPY2*-transgenic plants exhibited a stronger capacity to control stomatal aperture and reduce water loss under water stress. It is noteworthy that overexpression of *PeAPY1* or *PeAPY2* increased stomatal sensitivity to ABA. *PeAPY1* and *PeAPY2* enhanced the ABA action in stomatal control, including promoting closure in light and inhibiting opening of dark-adapted stomata transferred to light. The increased sensitivity to ABA is attributed to the modulation of eATP-mediated stomatal aperture in *PeAPY1-* and *PeAPY2*-transgenic plants. We postulate a speculative model of apyrase signaling in eATP- and ABA-regulated stomatal movements under drought stress.

## 2. Results

### 2.1. Leaf Water Loss and Stomatal Aperture under Water Stress

**Figure 1:**
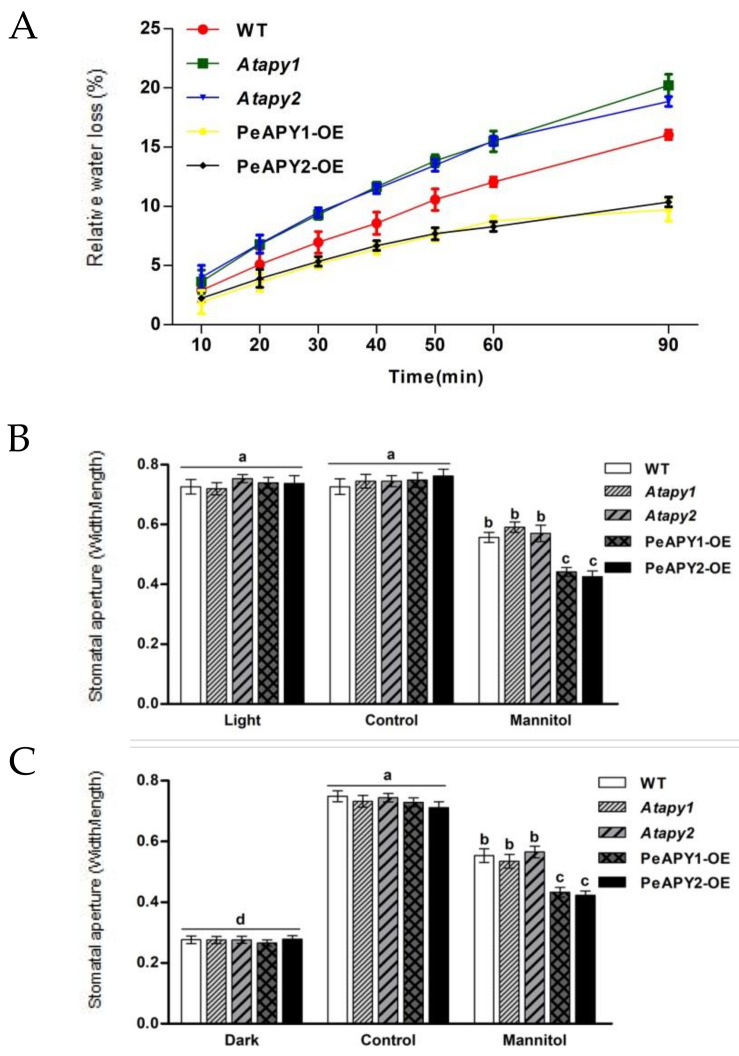
The relative water loss of leaves upon air exposure and the effect of mannitol on stomatal aperture in wild-type (WT), Arabidopsis loss-of-function mutants for *APY1* and *APY2* (*Atapy1* and *Atapy2*) and transgenic lines of *PeAPY1* and *PeAPY2* (PeAPY1-OE and PeAPY2-OE). (**A**) Leaves were excised from three-week-old seedlings and subjected to dehydration treatment in air under a light intensity of 150 mmol m^−2^ s^−1^. Water loss from the leaf surface was regularly measured during the period of 90 min air exposure. (**B**,**C**) Leaves isolated from three-week-old seedlings were incubated in MES-Tris buffer containing 50 mM KCl and 10 mM MES-Tris (pH 6.15) for 2 h in light (150 μmol m^−2^ s^−1^) (**B**) or in darkness (**C**). Thereafter, leaves were exposed to 0 (control) or 200 mM mannitol for 2 h in cool light (150 μmol m^−2^ s^−1^). Stomatal aperture was measured in continuously illuminated leaves (**B**) and dark-adapted plants transferred to light (**C**), respectively. Each column is the mean of three independent experiments, and error bars represent SE. Columns labeled with different letters, a, b, c and d, showed a significant difference at *p* < 0.05 between treatments and genotypes under conditions of continuous light (**B**) or dark-adapted leaves transferred to light (**C**).

The rate of water loss reflects plants’ ability to retain their water status under water stress [4,5,8,9]. The isolated leaves of *Atapy1* and *Atapy2*, the Arabidopsis loss-of-function mutants for *APY1* and *APY2*, exhibited a greater water loss rate than that of wild-type (WT) during the period of air exposure (90 min), while overexpression of *PeAPY1* or *PeAPY2* significantly lowered the water loss (Figure 1A). After exposure to an osmotic stress caused by 200 mM mannitol (2 h), *PeAPY1*- and *PeAPY2*-transgenic plants showed typical lower stomatal aperture compared with WT and mutants under light conditions, including continuous light and dark-adapted leaves transferred to light (Figure 1B,C). This indicates that the stronger water retention capacity exhibited by transgenic plants was, at least in part, due to the reduced stomatal conductance, although water loss will be much more dependent on epidermal properties rather than stomata in a prolonged air exposure.

### 2.2. Stomatal Response to ATP and Apyrase

**Figure 2:**
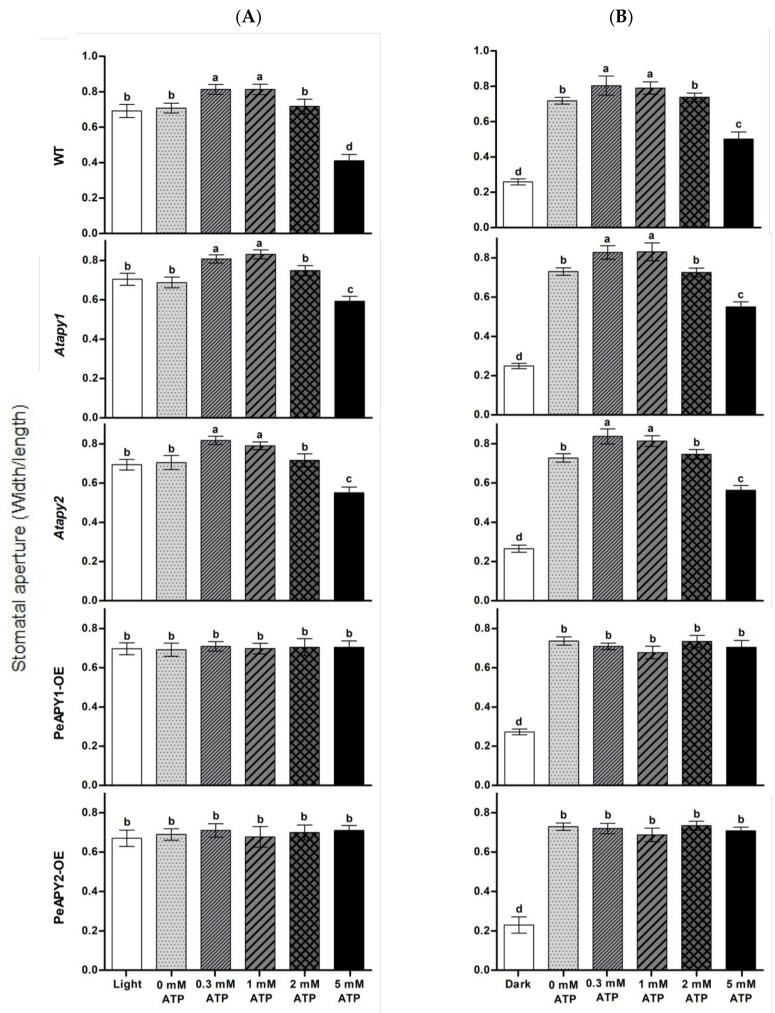
Effects of ATP on stomatal aperture in wild-type (WT), Arabidopsis loss-of-function mutants for *APY1* and *APY2* (*Atapy1* and *Atapy2*) and transgenic lines of *PeAPY1* and *PeAPY2* (PeAPY1-OE and PeAPY2-OE). Leaves from three-week-old seedlings were incubated in MES-Tris buffer containing 50 mM KCl and 10 mM MES-Tris (pH 6.15) in light (150 μmol m^−2^ s^−1^) (**A**) or in darkness (**B**). Thereafter, leaves were exposed to different concentrations of eATP (0, 0.3, 1.0, 2.0 or 5.0 mM) for 2 h in cool light (150 μmol m^−2^ s^−1^). Stomatal aperture was measured in continuously illuminated leaves (**A**) and dark-adapted leaves transferred to light (**B**), respectively. Each column is the mean of three independent experiments, and error bars represent SE. Columns labeled with different letters, a, b, c and d, showed a significant difference at *p* < 0.05 between treatments and genotypes under conditions of continuous light (**A**) or dark-adapted leaves transferred to light (**B**).

The dose effect of ATP on stomatal aperture was examined since eATP regulates stomatal aperture in a bi-phasic pattern [19]. Under continuous light conditions, exogenous application of ATP promoted opening in the WT at low concentrations of 300 and 1000 μM (Figure 2A). The nonhydrolyzable ATP analog, ATPγS (300 μM), increased opening in all tested genotypes (Supplementary Figure S1), which is similar to the promotion effect of low ATP concentration (300–1000 μM; Figure 2A). Our data are consistent with previous reports that show that hydrolyzable and nonhydrolyzable ATP analogs promote stomatal opening [19,20,21,22]. In contrast to ATP and ATPγS, applications of ADP and AMP at 300 μM did not significantly increase opening in WT, mutants and transgenic plants (Supplementary Figure S1). We noticed that the promotion of ATP on opening was not observed at 2.0 mM, and even a closure was induced at 5.0 mM (Figure 2A). This agrees with the finding by Clark et al., who found that a low dose of ATP (1.0 mM) induces stomatal opening, while application of high-dose ATP (1.5 mM) induced closure [21]. The Atapy1 and Atapy2 mutants showed a bi-phasic dose-response to applied nucleotides—a trend similar to WT in response to increasing ATP concentration (Figure 2A). In contrast to WT and the two mutants, the ATP-promoted opening at low concentrations and ATP-induced closure at high concentrations were both suppressed in transgenic plants overexpressing PeAPY1 or PeAPY2 (Figure 2A). 

The effects of ATP on light-induced opening were also tested when dark-adapted plants were transferred to light. The applied ATP nucleotides caused a bi-phasic response in WT, *Atapy1* and *Atapy2* mutants (Figure 2B). However, the increasing ATP had no significant effect on light-induced opening in transgenic lines, which is similar to the finding under continuous light conditions (Figure 2A,B).

**Figure 3:**
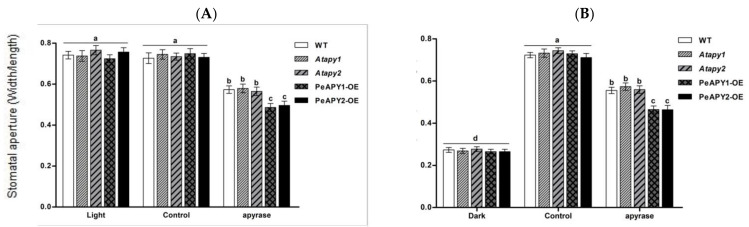
Effects of apyrase on stomatal aperture in wild-type (WT), Arabidopsis loss-of-function mutants for *APY1* and *APY2* (*Atapy1* and *Atapy2*) and transgenic lines of *PeAPY1* and *PeAPY2* (PeAPY1-OE and PeAPY2-OE). Leaves from three-week-old seedlings were incubated in MES-Tris buffer (pH 6.15) for 2 h in light (150 μmol m^−2^ s^−1^) (**A**) or in darkness (**B**). Thereafter, leaves were exposed to soluble potato apyrase (25 U mL^−1^) for 2 h in cool light (150 μmol m^−2^ s^−1^). Controls were treated without the addition of potato apyrase. Stomatal aperture was measured in continuously illuminated leaves (**A**) and dark-adapted plants transferred to light (**B**), respectively. Each column is the mean of three independent experiments, and error bars represent SE. Columns labeled with different letters, a, b, c and d, showed a significant difference at *p* < 0.05 between treatments and genotypes under conditions of continuous light (**A**) or dark-adapted leaves transferred to light (**B**).

Potato apyrase, which can hydrolyze ATP [20,21], was used to testify to its effect on stomatal aperture in continuously illuminated leaves (Figure 3A) and dark-adapted leaves transferred to light (Figure 3B). The applied apyrase significantly reduced stomatal aperture in all tested lines, but with a more pronounced inhibition in *PeAPY1*- and *PeAPY2*-transgenic plants (Figure 3A,B).

### 2.3. Stomatal Response to ABA and ATP Trap

**Figure 4:**
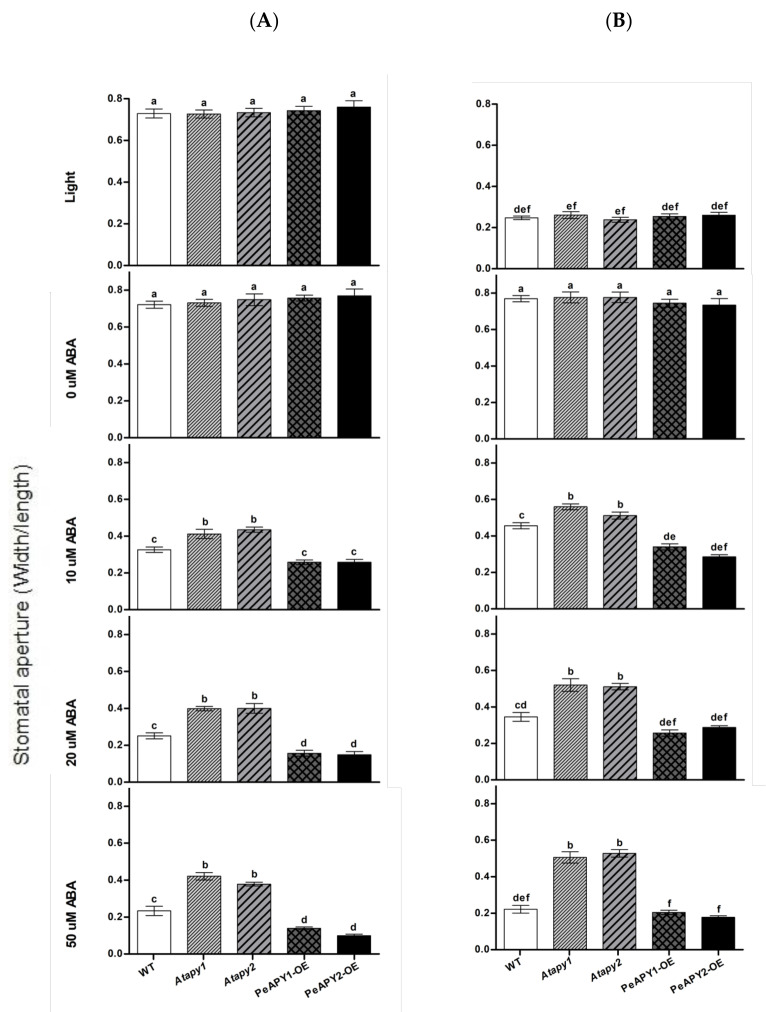
Effects of ABA on stomatal aperture in wild-type (WT), Arabidopsis loss-of-function mutants for *APY1* and *APY2* (*Atapy1* and *Atapy2*) and transgenic lines of *PeAPY1* and *PeAPY2* (PeAPY1-OE and PeAPY2-OE). Leaves from three-week-old seedlings were incubated in MES-Tris buffer (pH 6.15) for 2 h in light (150 μmol m^−2^ s^−1^) (**A**) or in darkness (**B**). Thereafter, leaves were exposed to different concentrations of ABA (0, 10, 20 or 50 μM) for 2 h in cool light (150 μmol m^−2^ s^−1^). Stomatal aperture was measured in continuously illuminated leaves (**A**) and dark-adapted plants transferred to light (**B**), respectively. Each column is the mean of three independent experiments, and error bars represent SE. Columns labeled with different letters, a, b, c, d, e and f, showed a significant difference at *p* < 0.05 between treatments and genotypes under conditions of continuous light (**A**) or dark-adapted leaves transferred to light (**B**).

The effect of ABA on stomatal aperture was testified in apyrase genes transferred plants, since ABA is crucial in mediating stomatal responses [4,27]. In the absence of ABA, the tested genotype showed no significant difference in the stomatal aperture under either light (Figure 4A) or dark conditions (Figure 4B). ABA-induced stomatal closure in light was observed at all tested concentrations, 10, 20 and 50 μM (Figure 4A). Of note, the function of ABA was more pronounced in *PeAPY1*- or *PeAPY2*-overexpressed plants (Figure 4A). In contrast to these transgenic lines, the loss-of-function mutants Atapy1 and Atapy2 exhibited lower sensitivity to ABA (Figure 4A). This is in agreement with Clark et al., who found that RNAi suppression of APY1 in an apy2 single knockout resulted in more open stomata upon ABA exposure [19]. 

ABA-inhibited stomatal opening was also tested after dark-adapted plants were transferred to light. The light-induced opening was inhibited by ABA in all tested genotypes, regardless of ABA concentrations (Figure 4B). Compared to the WT, the ABA-inhibited opening was enhanced in *PeAPY1*- or *PeAPY2*-transgenic plants, but less pronounced in the *Atapy1* and *Atapy2* mutants (Figure 4B).

**Figure 5:**
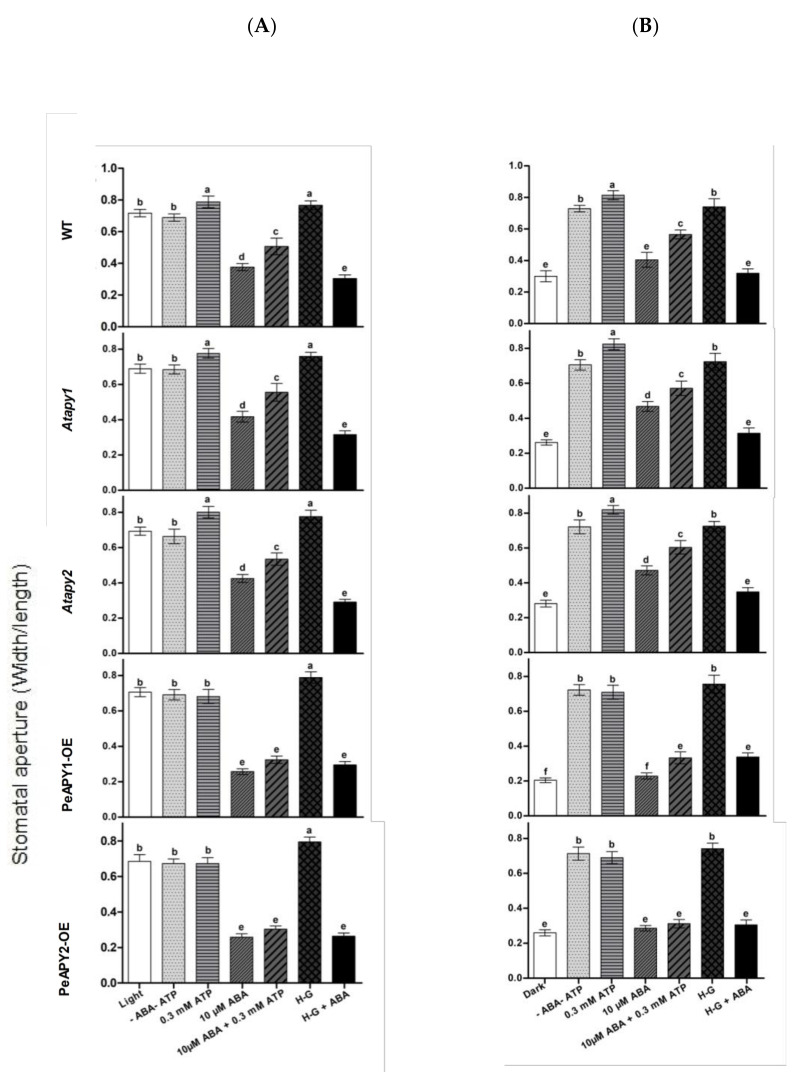
Effects of ATP and ATP trap on ABA-regulated stomatal aperture in wild-type (WT), Arabidopsis loss-of-function mutants for *APY1* and *APY2* (*Atapy1* and *Atapy2*) and transgenic lines of *PeAPY1* and *PeAPY2* (PeAPY1-OE and PeAPY2-OE). Leaves from three-week-old seedlings were incubated in MES-Tris buffer containing 50 mM KCl and 10 mM MES-Tris (pH 6.15) for 2 h in light (150 μmol m^−2^ s^−1^) (**A**) or in darkness (**B**). Thereafter, leaves were exposed to 0.3 mM eATP or H-G trap that comprised 50 mM glucose and 100 U mL^−1^ hexokinase for 2 h in cool light (200 μmol m^−2^ s^−1^) in the presence and absence of ABA (10 μM). Controls (-ATP-ABA) were treated without the addition of ATP, H-G or ABA. Stomatal aperture was measured in continuously illuminated leaves (**A**) and dark-adapted plants transferred to light (**B**), respectively. Each column is the mean of three independent experiments, and error bars represent SE. Columns labeled with different letters, a, b, c, d, e and f, showed a significant difference at *p* < 0.05 between treatments and genotypes under conditions of continuous light (**A**) or dark-adapted leaves transferred to light (**B**).

Moreover, the interaction between ATP and ABA on stomatal aperture was examined in our study. The low concentration of ATP (300 μM) was found to counteract with ABA (10 μM) in mediating stomatal aperture (Figure 5). The ABA-induced closure (Figure 5A) and ABA-inhibited opening (Figure 5B) in WT, *Atapy1* and *Atapy2* mutants were both reduced by the ATP application. However, the inhibition of ATP on ABA action was not so pronounced in *PeAPY1*- and *PeAPY2*-transgenic plants as that observed in WT and mutants, since these transgenic plants had exhibited typically high sensitivity to ABA (Figure 5A,B).

To confirm the distinct inhibitory effect of low ATP on ABA, eATP was depleted with a trap that comprised 50 mM of glucose and 100 units/mL of hexokinase (H-G) [37]. In the absence of ABA, H-G-treated leaves showed high stomatal aperture in continuously illuminated leaves (Figure 5A) and dark-adapted leaves transferred to light (Figure 5B). When 10 μM of ABA was applied, the ABA-induced closure and ABA-inhibited opening were both enhanced by H-G in WT, *Atapy1* and *Atapy2* mutants (Figure 5A,B). However, the enhancement of H-G was less pronounced in the transgenic lines that were hypersensitive to ABA (Figure 5A,B). Our data showed that depleting eATP with the H-G system strengthened the control of ABA on stomatal movements.

### 2.4. Purinoceptor Inhibitors Mediate ABA-Regulated Stomatal Movement

**Figure 6:**
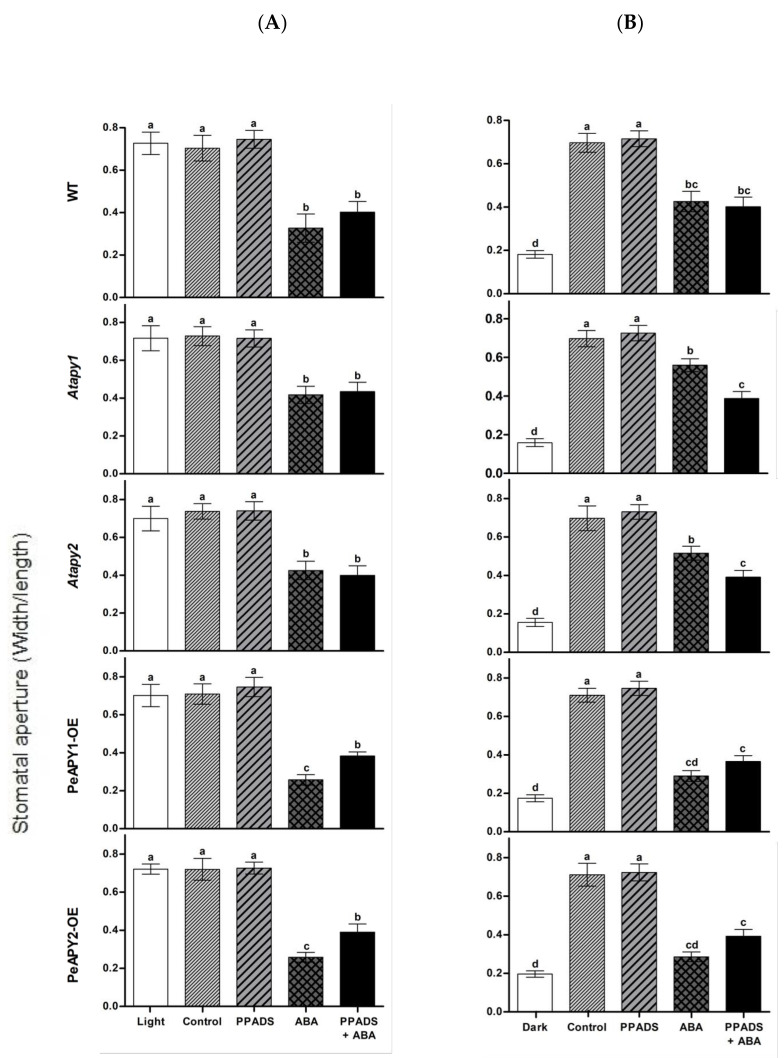
Effects of purinergic receptor antagonist, PPADS, on ABA-regulated stomatal aperture in wild-type (WT), Arabidopsis loss-of-function mutants for *APY1* and *APY2* (*Atapy1* and *Atapy2*) and transgenic lines of *PeAPY1* and *PeAPY2* (PeAPY1-OE and PeAPY2-OE). Leaves from three-week-old seedlings were incubated in MES-Tris buffer containing 50 mM KCl and 10 mM MES-Tris (pH 6.15) for 2 h in light (150 μmol m^−2^ s^−1^) (**A**) or in darkness (**B**). Thereafter, leaves were exposed to 100 μM PPADS for 2 h in cool light (150 μmol m^−2^ s^−1^) in the presence and absence of ABA (10 μM). Controls were treated without the addition of inhibitor or ABA. Stomatal aperture was measured in continuously illuminated leaves (**A**) and dark-adapted plants transferred to light (**B**), respectively. Each column is the mean of three independent experiments, and error bars represent SE. Columns labeled with different letters, a, b, c and d, showed a significant difference at *p* < 0.05 between treatments and genotypes under conditions of continuous light (**A**) or dark-adapted leaves transferred to light (**B**).

Antagonist of animal purinoceptor, pyridoxalphosphate-6-azo-phenyl-2′,4′-disulfonic acid (PPADS, 100 μM), is shown to partially block the ABA-induced stomatal closure [19]. To testify the effect of purinoceptor inhibitors on ABA-regulated stomatal movements, WT, Arabidopsis mutants and transgenic lines were treated with suramin or PPADS under continuous light or by transferring dark-adapted leaves to light. The antagonists of animal purinoceptors alone had no effect on stomatal apertures at tested concentrations of 50, 100, 200 or 300 μM (Supplementary Figure S2A,B). In the presence of ABA, suramin or PPADS significantly reduced the ABA-induced stomatal closure in *PeAPY1*- and *PeAPY2*-transgenic plants, while the blocking of purinoceptor inhibitors on ABA signaling was less pronounced in WT and mutants (Figure 6A,B; Supplementary Figure S3A,B).

### 2.5. ABA-Induced Expression of NADPH Oxidase Genes

**Figure 7:**
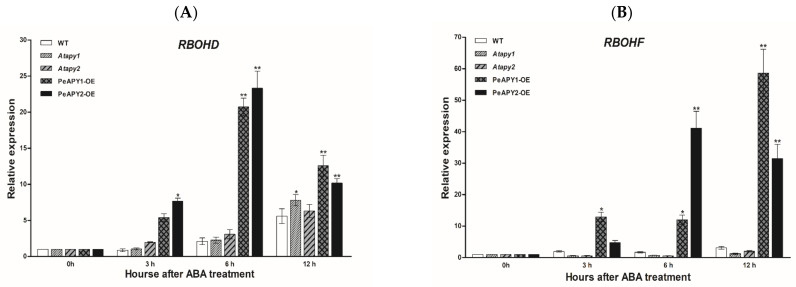
Effects of ABA on *AtRBOHD* and *AtRBOHF* expression in wild-type (WT), Arabidopsis loss-of-function mutants for *APY1* and *APY2* (*Atapy1* and *Atapy2*) and transgenic lines of *PeAPY1* and *PeAPY2* (PeAPY1-OE and PeAPY2-OE). (**A**) *AtRBOHD* expression. (**B**) *AtRBOHF* expression. Ten-day-old seedlings were exposed to ABA (0 or 10 μM) in light (150 μmol m^−2^ s^−1^), and pH was adjusted to 5.7–5.8 when ABA was added into the nutrient solution. Expression of *AtRBOHD* and *AtRBOHF* were examined after 0, 3, 6 and 12 h of ABA treatment. The Arabidopsis *AtACTIN2* was used as the internal control. Primers designed to target *AtRBOHD*, *AtRBOHF* and *AtACTIN2* genes are shown in Supplementary Table S2. Each column is the mean of three independent experiments, and error bars represent SE. Columns labeled with asterisks showed significant differences between transgenic lines, WT and mutants at each sampling time: * *p* < 0.05, ** *p* < 0.01.

In this study, transcription of *AtRBOHD* and *AtRBOHF* genes, encoding Arabidopsis NADPH oxidases, was examined since ROS contributes to the ABA-induced stomatal closure [19]. Under light conditions, the transcripts of *AtRBOHD* and A*tRBOHF* genes remained at low levels and were similar in the tested genotypes (Figure 7A,B). ABA upregulated the expression of *AtRBOHD* and *AtRBOHF* during the period of treatment (3, 6, 12 h), and a pronounced effect was observed in transgenic plants overexpressing *PeAPY1* and *PeAPY2* (Figure 7A,B).

## 3. Discussion

Water deficit usually induces ABA synthesis, which contributes to reducing water loss [4,27]. In this study, *PeAPY1*- and *PeAPY2*-transgenic plants increased water retention capacity partly due to low stomatal aperture (Figure 1). It is interesting to find that *PeAPY1*- and *PeAPY2*-transgenic lines showed an increased sensitivity to ABA (Figure 4), which helps to retain water status under stress conditions [33,34,35]. Our data show that *P. euphratica* apyrases mediate eATP and ABA signaling to retain water status under drought stress.

*P. euphratica* apyrases mediate eATP-regulated stomatal aperture under light conditions. Exogenous application of ATP at low concentrations (0.3 and 1.0 mM) could promote stomata opening of wild-type, but high concentrations of ATP (>2 mM) caused stomatal closure (Figure 2). This agrees with previous reports that showed that applying ATP in hydrolyzable and nonhydrolyzable forms caused a bi-phasic dose-response in *A. thaliana* and *V. faba* [19,20,21,22]. However, the ATP-promoted opening at low concentrations and the closure induced by high ATP concentrations were both suppressed in transgenic plants overexpressing *PeAPY1* or *PeAPY2* (Figure 2). This suggests that *P. euphratica* apyrases contributed to the control of eATP-regulated stomatal movements not only at low but also at high concentrations. The experimental evidence and possible explanations are provided below.

*P. euphratica* apyrases inhibited low eATP-promoted opening. The low ATP (0.3 and 1.0 mM)-stimulated stomata opening was inhibited in *PeAPY1*- and *PeAPY2*-overexpressed plants under light conditions, including continuous light and dark-adapted stomata transferred to light (Figure 2). This was due to the increased enzymatic activity on hydrolyzing purine nucleotides [26]. The introduced *P. euphratica* apyrases in transgenic plants reduced eATP levels, thus inhibiting ATP-promoted opening. In accordance, the applied potato apyrase, which would decrease endogenous eATP levels [20,21], significantly suppressed stomatal aperture in all tested lines (Figure 3). Similarly, application of soluble potato apyrase blocked stomatal opening of *A. thaliana* in the light [20].

We noticed that the apyrase inhibition was more pronounced in *PeAPY1* and *PeAPY2*-transgenic plants (Figure 3), suggesting the combination effects of ectopic (*PeAPY1* and *PeAPY2*) and endogenous Arabidopsis ectoapyrases on the ATP control. These findings are also consistent with the results obtained by external applied chemical inhibitors of apyrase enzymes. External application of chemical apyrase inhibitors, such as NGXT 191 (1.5 μg/mL) and apyrase inhibitor #13 (1.5 μg/mL), which moderately inhibited ectoapyrase activity, would increase endogenous eATP levels, resulting in stomatal opening in darkness [21]. In our study, the loss-of-function *APY1* and *APY2* mutants, which might have reduced ATP hydrolyzing activity, would retain eATP to stimulate opening in light (Figure 2). This is in accordance with the finding that RNAi suppression of apyrase expression leads to increased stomatal aperture in response to light [19]. Therefore, *PeAPY1*- or *PeAPY2*-overexpressed plants could effectively hydrolyze the external applied eATP, thus reducing the ATP stimulation effect in opening.

*P. euphratica* apyrases inhibited high eATP-promoted closure. Application of high doses of ATP (>2 mM) caused stomatal closure in WT, *Atapy1* and *Atapy2* (Figure 2) [19,21]. Evidence was found with pharmacological agents, showing that treatment of epidermal peels with a high dose of apyrase inhibitor NGXT 191, 7.5 mg/mL, which would cause naturally occurring levels of eATP to remarkably increase to high levels, induced stomatal closure in light [19]. However, *PeAPY1*- and *PeAPY2*-overexpressed plants kept the stomata open under high eATP in light (Figure 2). This indicates that the transgenic plants possessed the ability to hydrolyze ATP [26], which could reduce the high eATP-stimulated closure (Figure 2). In guard cell protoplasts, APY is thought to control eATP at low concentrations to promote the opening since APY expression rises quickly when these cells are moved from darkness into light [19]. In our study, the application of NGXT 191 on *PeAPY1*- and *PeAPY2*- transgenic plants was not attempted because the strong inhibitor of apyrase activity in Arabidopsis and potato [38,39] did not significantly inhibit apyrase in *P. euphratica* [26]. Taken together, our data showed that *PeAPY1* and *PeAPY2* could play a role in regulating stomatal aperture in light, i.e., inhibiting the low eATP-promoted opening and high ATP-induced closure.

It is worth noting that *P. euphratica* apyrases mediate ABA-regulated stomatal closure. ABA applied at concentrations of 10–50 μM was shown to induce stomatal closure in light and inhibit stomatal opening when dark-adapted leaves were exposed to light (Figure 4). We observed that low eATP counteracted ABA action in the regulation of stomatal aperture (Figure 5). The experimental evidence is briefly listed as follows. (a) The direct addition of 300 μM ATP, which promoted opening (Figure 2), could limit the role of ABA (10 μM) in stomatal closure (Figure 5). (b) When endogenous eATP was depleted with a H-G trap system, the ABA-induced closure and ABA-inhibited opening were enhanced in WT, *Atapy1* and *Atapy2* mutants (Figure 5). (c) The loss function of either *Atapy1* or *Atapy2* in Arabidopsis, which would retain endogenous eATP levels by reducing apyrase, facilitated eATP-promoted stomatal opening, and thus reduced the ABA action in promoting closure (Figure 4). Similarly, RNAi suppression of *APY1* in an *apy2* single knockout results in increased stomatal apertures compared with wild-type under ABA treatment [19]. (d) Overexpression of *PeAPY1* or *PeAPY2*, which could reduce eATP levels, enhanced both the ABA-induced closure and ABA-inhibited opening (Figure 4). Our finding of distinct action between ATP (300 μM) and ABA (10 μM) in the stomatal regulation is not contrary to their additive effect in closing stomata, where concentrations of ABA, 0.1 μM, and ATPγS, 75 μM, were too low on their own to have an apparent effect on stomatal closure [21]. Therefore, overexpression of either *PeAPY1* or *PeAPY2* in Arabidopsis would decrease endogenous eATP levels by elevated apyrase [26], which blocked eATP-promoted stomatal opening in the light, thus enhancing the ABA action in closure. Clark et al. have shown that added potato apyrase could block ABA-induced stomatal closure, indicating the role of ABA-induced release of eATP in stomatal aperture control [21]. However, we found that *PeAPY1* and *PeAPY2* overexpression enhanced the ABA-induced closure (Figure 4). The inconsistent results might be due to the greater capacity of introduced apyrases in eATP control in transgenic plants. *P. euphratica* apyrase, for example, apyrase 2, has a Golgi localization [26] similar to the APY1 and APY2 in Arabidopsis [40,41,42]. It is suggested that apyrase could indirectly control the concentration of ATP through the regulation of the luminal eATP in secretory vesicles derived from the Golgi [25]. Thus, it can be inferred that *P. euphratica* apyrases could strictly control ATP via their activity in both the lumen of the Golgi and on the outer face of the PM [19,26]. The external potato apyrase could hydrolyze the ATP in the ECM but may not suppress the release of ATP from secretory vesicles. As a result, the remaining eATP would promote opening, thus blocking the ABA action in closure (Figure 5). Hence, it is possible that *P. euphratica* apyrases effectively controlled eATP to diminish its promotion in opening (Figure 2), which consequently enhanced ABA-induced stomatal closure in transgenic plants. We observed that ABA-induced stomatal closure was significantly blocked by suramin or PPADS in transgenic plants, but was less evident in WT and mutants (Figure 6; Supplementary Figure S3). This suggests that in transgenic plants, the downstream signaling components initiated by eATP may play a critical role in the complex signaling pathway mediated by ABA [19]. In addition, ABA substantially increased transcription of NADPH oxidase genes, *AtRBOHD* and *AtRBOHF*, in transgenic plants (Figure 7). It is suggested that the ATP receptor, DORN1, can cause direct phosphorylation of RBOHD, resulting in elevated production of reactive oxygen species and stomatal closure [23]. Therefore, the ABA-elicited ROS contributed to stomatal closure in transgenic Arabidopsis overexpressing *PeAPY1* or *PeAPY2*.

## 4. Materials and Methods

### 4.1. P. euphratica Culture Conditions

One-year-old seedlings of *P. euphratica,* obtained from Xinjiang Uygur Autonomous Region (China), were planted in pots (10 L) containing loam and sand (1:1) and cultured in a greenhouse of Beijing Forestry University. The temperature was 20–25 °C, with a 16 h photoperiod (7:00–23:00) and a photosynthetically active radiation of 150–300 μmol m^−2^s^−1^. Potted plants were irrigated according to evaporation requirements and fertilized with 1 L of full-strength Hoagland nutrient solution every 2 weeks [43,44].

Callus induction from the upper shoots of *P. euphratica* and subculture were performed as previously described [37,45,46,47]. The callus was cultured on MS (Murashige and Skoog) solid medium (2.5% sucrose, pH 6.0) containing 0.50 mg L^−1^ of 6-benzyl adenine (BA) and 0.50 mg L^−1^ of naphthaleneacetic acid (NAA). The callus was cultured in the dark at 25 °C and sub-cultured every 20 days.

### 4.2. Cloning of PeAPY1 and PeAPY2 Genes

Total RNA was extracted from the callus of *P. euphratica* using TRIzol reagent (Invitrogen, Carlsbad, CA, USA), and then the total RNA of *P. euphratica* was reverse transcribed by Oligo dT (Promega, Madison, WI, USA) to synthesize cDNA, which was used as a PCR template [26,33,34]. Primers were designed based on the homologous apyrase gene sequence of *Populus trichocarpa* (Pt; reference sequence number XM_002-325360 on NCBI). The primers for (i) *PeAPY1* were 5′-ATGAATAATAAGTTGAAG CTGATGGGCT-3′ (upstream) and 5′-CTACTTCAGAAA TGATGCACTGCTAGGTG-3′ (downstream), and for (ii) *PeAPY2* were 5′-ATGAAACGA CCTGGTTTGCGAC-3′ (upstream) and 5′-TTATGCTGGTGATGACACAGCCTC-3′ (downstream), respectively. The full length of *PeAPY1* and *PeAPY2* genes was amplified by PCR at 56 °C for 90 s and 35 cycles. The amplified products were then purified and recombined into pMD18-T vector (Takara, Kusatsu, Japan), and then the recombinant product was transformed into *Escherichia coli* Top10 competent cells (Invitrogen, Carlsbad, CA, USA). Single colonies grown on ampicillin-resistant LB medium were picked and cultured in liquid medium, and the bacterial cells were identified by PCR and sequenced to obtain the full-length sequence of *P. euphratica APY1* and *APY2* [26,33,34].

### 4.3. Sequence and Phylogenetic Analyses

We compared the amino acid sequences of apyrases from different plant species with ClustalW (http://www.genome.jp/tools/clustalw/, accessed on 18 August 2020) (EMBL-EBI, Hinxton, Cambridgeshire, UK) (Supplementary Figure S4). Multiple sequence alignment of apyrases showed that PeAPY1 and PeAPY2 are homologous to *P. tricocarpa* APY (Supplementary Figure S4) [26]. Phylogenetic tree of apyrases were constructed by the neighbor-joining method with 1000 bootstrap replicates using MEGA 5.2 software (http://www.megasoftware.net/index.php, accessed on 18 August 2020) (Center for Evolutionary Medicine and Informatics, Tempe, AZ, USA) (Supplementary Figure S5). The GeneBank accession numbers for plant apyrases and the *Arabidopsis* homolog locus are listed in Supplementary Table S1. The constructed phylogenetic dendrogram shows the evolutionary conservation of PeAPYs with other APYs (Supplementary Figure S5) [26].

### 4.4. Construction and Screening of PeAPY1- and PeAPY2-Transgenic Lines

The transformation of *PeAPY1* and *PeAPY2* into Arabidopsis has been described by Tan et al. [34] and Deng et al. [26]. In brief, the full-length genes of *PeAPY1* and *PeAPY2* were cloned into the expression vector pK7WG2D (Flanders Interuniversity Institute of Biotechnology, Ghent University, Ghent, Belgium) containing the 35S promoter to obtain recombinant plasmids pK7WG2D-*PeAPY1* and pK7WG2D-*PeAPY2*, respectively. The recombinant plasmid was transformed into *Agrobacterium tumefaciens* strain GV3101. Then, the recombinant plasmid and the empty vector were separately transformed into *Arabidopsis thaliana* wild-type by dip flower infection. The obtained transgenic Arabidopsis seeds were screened on kanamycin-containing medium, the positive plants were transplanted into the soil for culture and the seeds were placed on the kanamycin-containing plate. The kanamycin-resistant plants in the T2 generation were transplanted into the soil to continue growth, homozygous plants were identified, and homozygous seeds were harvested for subsequent experiments. The expression levels of *PeAPY1* and *PeAPY2* were examined by real-time quantitative PCR (RT-qPCR) and semi-quantitative RT-PCR [26]. Seeds of the Arabidopsis loss-of-function mutants for *APY1* and *APY2* (*Atapy1* and *Atapy2*) were obtained from the Arabidopsis Biological Resource Center (ABRC, Ohio State University, Columbus, OH, USA).

Arabidopsis seeds of WT, *Atapy1*, *Atapy2* and transgenic lines, PeAPY1-OE and PeAPY2-OE (T3 generation), were sterilized with 1% sodium hypochlorite for 7–10 min, washed 3–5 times with sterile water and sown in sterile 1/2 MS medium supplemented with 1% sucrose, 3 g L^−1^ plant gel, pH 5.8. After 4 °C low-temperature stratification treatment, seeds were germinated and grown in a climate chamber. The chamber was controlled at a temperature of 22/21 °C (day/night) and the relative humidity was 70%. The light intensity was 150 μmol m^−2^ s^−1^ with a photoperiod of 16 h/8 h (light/dark). Ten-day-old seedlings grown in the medium were transferred to nursery soil for a further 20-day culture in a growth room under a long-day photoperiod (16 h of light and 8 h of dark) at 150 μmol m^−2^ s^−1^. The temperature was 22 °C with 50–70% relative humidity (RH).

### 4.5. Water Loss Measurement

Leaf water loss was examined to compare the difference in maintaining water status between wild-type, mutant and transgenic lines. Immediately after the upper mature leaves (the third-fifth from the tip) were excised from three-week-old seedlings, the fresh weight (FW_0_) was obtained. Thereafter, leaf samples were placed on the laboratory bench under a light intensity of 150 μmol m^−2^ s^−1^, and water loss from the leaf surface was regularly measured during the period of 90 min air exposure [48]. Air temperature was 25 °C and RH was 50–60%. The relative leaf water loss (RWL) was calculated as follows:

RWL (%) = (FW_0_ − FW)/(FW_0_ − DW) × 100

where FW is the leaf fresh weight during the period of air exposure, and DW represents the dry weight.

### 4.6. Mannitol Treatment

In a hyperosmotic treatment, leaves isolated from three-week-old plants of the tested genotypes were subjected to MES-Tris buffer containing 50 mM KCl and 10 mM MES-Tris (pH 6.15) [20] for 2 h in light (150 μmol m^−2^ s^−1^) or in darkness. Thereafter, leaves were exposed to 200 mM mannitol solution for 2 h in cool light (150 μmol m^−2^ s^−1^) and stomatal aperture was measured as described below.

### 4.7. Stomatal Aperture Measurements

Upper mature leaves (the third-fifth from the tip) were sampled from three-week-old seedlings and incubated in MES-Tris buffer containing 50 mM KCl and 10 mM MES-Tris (pH 6.15) [20]. These leaves were subjected to the following treatments: (i) Continuous illumination: Leaves were irradiated with a cold light source (150 μmol m^−2^s^−1^) for 2 h to completely open the stomata. Thereafter, ATP in hydrolyzable and nonhydrolyzable forms, ADP, AMP, apyrase, ABA and pharmacological reagents (PPADS, suramin) were added to continue the illumination for 2 h. (ii) Dark to light transferring: Leaves were incubated in MES-Tris buffer in darkness for 2 h to completely close the stomata. Then, the leaves were exposed to the same agents as applied in (i) and illuminated with cool light (150 μmol m^−2^ s^−1^) for 2 h to induce stomatal opening. In this study, six series of experiments were carried out, as described below.

Series 1 Nucleotides Treatment: Leaves sampled from WT, *Atapy1*, *Atapy2*, *PeAPY1*- and *PeAPY2*-transgenic lines (PeAPY1-OE1 and PeAPY2-OE2, T3 generation) were incubated in MES-Tris buffer containing 50 mM KCl and 10 mM MES-Tris, pH 6.15 [20], for 2 h in cool light (150 μmol m^−2^ s^−1^) or in darkness, as described above. Thereafter, these leaves were exposed to ATP (0, 0.3, 1.0, 2.0 and 5.0 mM), ADP (0.3 mM), AMP (0.3 mM) or ATPγS (0.3 mM) for 2 h in cool light (150 μmol m^−2^ s^−1^).

Series 2 Apyrase Treatment: Leaves from WT, *Atapy1*, *Atapy2*, PeAPY1-OE1 and PeAPY2-OE2 were incubated in MES-Tris buffer (pH 6.15) for 2 h in light or in darkness. Then, these leaves were exposed to soluble potato apyrase (25 U mL^−1^) [20] for 2 h in cool light (150 μmol m^−2^ s^−1^).

Series 3 ABA Treatment: Leaves of WT, *Atapy1*, *Atapy2*, PeAPY1-OE1 and PeAPY2-OE2 were incubated in MES-Tris buffer (pH 6.15) for 2 h in light or in darkness. Thereafter, leaves were exposed to different concentrations of ABA (0, 10, 20 or 50 μM) for 2 h in cool light (150 μmol m^−2^ s^−1^).

Series 4 ATP and ABA Treatment: Leaves from WT, *Atapy1*, *Atapy2*, PeAPY1-OE1 and PeAPY2-OE2 were incubated in MES-Tris buffer (pH 6.15) for 2 h in light or in darkness. Then, these leaves were exposed to 0.3 mM eATP for 2 h in cool light in the presence and absence of ABA (10 μM).

Series 5 ATP Trap and ABA Treatment: Leaves of WT, *Atapy1*, *Atapy2*, PeAPY1-OE1 and PeAPY2-OE2 were incubated in MES-Tris buffer (pH 6.15) for 2 h in light or in darkness. Thereafter, these leaves were exposed to H-G trap that comprised 50 mM of glucose and 100 units/mL of hexokinase [37] for 2 h in cool light in the presence and absence of ABA (10 μM).

Series 6 Purinergic Receptor Antagonist Treatment: Dose effect of animal purinergic receptor antagonists, suramin and PPADS, on stomatal aperture, was examined. Leaves from three-week-old seedlings were incubated in MES-Tris buffer containing 50 mM KCl and 10 mM MES-Tris for 2 h in light or in darkness. Then, leaves were exposed to different concentrations of suramin or PPADS (0, 50, 100, 200 or 300 μM) [37] for 2 h in cool light (150 μmol m^−2^ s^−1^).

The interaction between ABA and purinergic receptor antagonists was also testified. Upper mature leaves were incubated in MES-Tris buffer containing 50 mM KCl and 10 mM MES-Tris for 2 h in light or in darkness. Thereafter, these leaves were exposed to 100 μM suramin or PPADS for 2 h in cool light (200 μmol m^−2^ s^−1^) in the presence and absence of ABA (10 μM).

Following treatments of Series 1–6, stomatal aperture was measured in continuous light-treated leaves and dark-adapted plants transferred to light. The abaxial epidermis were taken from leaves and photographed at 40× with a Leica microscope (Leica Microsystems GmbH, Wetzlar, Germany). The length and width of the pores were measured using Image J software (National Institutes of Health, Bethesda, MD, USA), and the opening of the pores was expressed by the aspect ratio. Each treatment was randomly selected from 50–80 pores for calculation. The experiment was repeated at least three times, independently.

### 4.8. Quantitative Real-Time PCR

Ten-day-old seedlings of all genotypes, WT, *Atapy1*, *Atapy2*, PeAPY1-OE1 and PeAPY2-OE2, were transferred to 1/2 MS medium supplemented without or with 10 µM ABA for 12 h. Gene-specific primers were used to analyze the expression level of *AtRBOHD* and *AtRBOHF* by real-time quantitative PCR (RT-qPCR) [49]. Total RNA was isolated from leaves of WT, *Atapy1*, *Atapy2*, PeAPY1-OE1 and PeAPY2-OE2 using the EASYspin Plus Plant RNA Kit (Aidlab Biotech, Beijing, China) and Trizol reagent (Invitrogen, Carlsbad, California, USA), according to the manufacturer’s instructions. The RNase-free DNase (Promega, Madison, Wisconsin, USA) was used to remove DNA in the isolated RNA. Then, 1 μg RNA was used for reverse transcription with M-MLV reverse transcriptase (Promega, Madison, Wisconsin, USA) and an oligo (dT) primer (Promega, Madison, Wisconsin, USA). The products were used as the template for RT-qPCR. The Arabidopsis *AtACTIN2* was used as the internal control. Primers designed to target *AtRBOHD*, *AtRBOHF* and *AtACTIN2* genes are shown in Supplementary Table S2.

RT-qPCR was performed in a total volume of 20 µL, containing 0.5 µL forward and reverse primers (10 µM), 100 ng template (2 µL), 10 µL 2× SYBR mix and 7 µL RNase-free ddH_2_O [49]. The PCR running conditions for RT-qPCR were: 95 °C for 10 min, followed by 35 cycles of denaturation at 95 °C for 10 s, 55 °C for 30 s and extension at 72 °C for 30 s, and finally at 72 °C for 10 min. The RT-qPCR analysis was performed based on the instructions provided for the Applied Biosystems 7500 real-time PCR system (Applied Biosystems, Carlsbad, CA, USA). Each sample was repeated three times. The Ct value of the target gene was expressed as 2^−ΔΔCT^ based on the method of Livak and Schmittgen [50].

### 4.9. Data Analysis

All experimental data were subjected to statistical analysis using SPSS version 19.0 (IBM Corporation, Armonk, NY, USA). Unless otherwise stated, differences were considered significant at *p* < 0.05.

## 5. Conclusions

**Figure 8:**
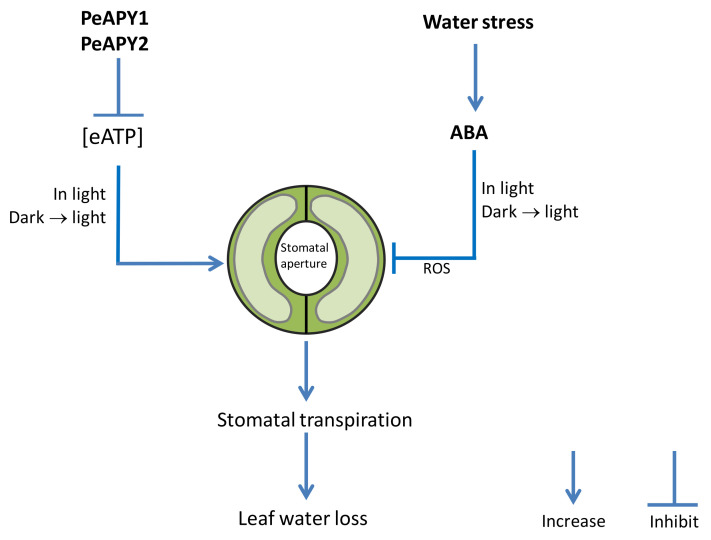
A schematic model showing that *Populus euphratica* apyrases increase stomatal sensitivity to ABA in Arabidopsis.

We proposed a signaling pathway in which *P. euphratica* apyrases mediate eATP- and ABA-induced stomatal aperture in transgenic Arabidopsis. As shown in Figure 8, extracellular ATP stimulates stomata opening under conditions of continuous light and dark-adapted stomata transferred to light. PeAPY1 and PeAPY2 strictly controlled eATP, which can (i) diminish its promotion in opening, and (ii) enhance ABA action in closure since eATP counteracts ABA in the regulation of stomatal aperture. Consequently, *PeAPY1*- and *PeAPY2*-transgenic plants reduced transpiration, thus increasing water-retaining capacity under water stress. The model predicts that ABA-elicited ROS contributed to stomatal closure since ABA induced transcription of *AtRBOHD* and *AtRBOHF* genes in transgenic Arabidopsis overexpressing *PeAPY1* or *PeAPY2*.

## Data Availability

The data presented in this study are available in the article and Supplementary Materials.

## Supplementary Materials

The following are available online at https://www.mdpi.com/article/10.3390/ijms22189892/s1.
