# Supplementary material for: Populus euphratica Apyrases Increase Drought Tolerance by Modulating Stomatal Aperture in Arabidopsis
Source: Int J Mol Sci. 2021 Sep 13;22(18):9892. doi: 10.3390/ijms22189892 (PMC8468604; doi:10.3390/ijms22189892)
Supplement: Supplementary file 1 [file ijms-22-09892-s001.zip › Figure S3_corrected.pdf]

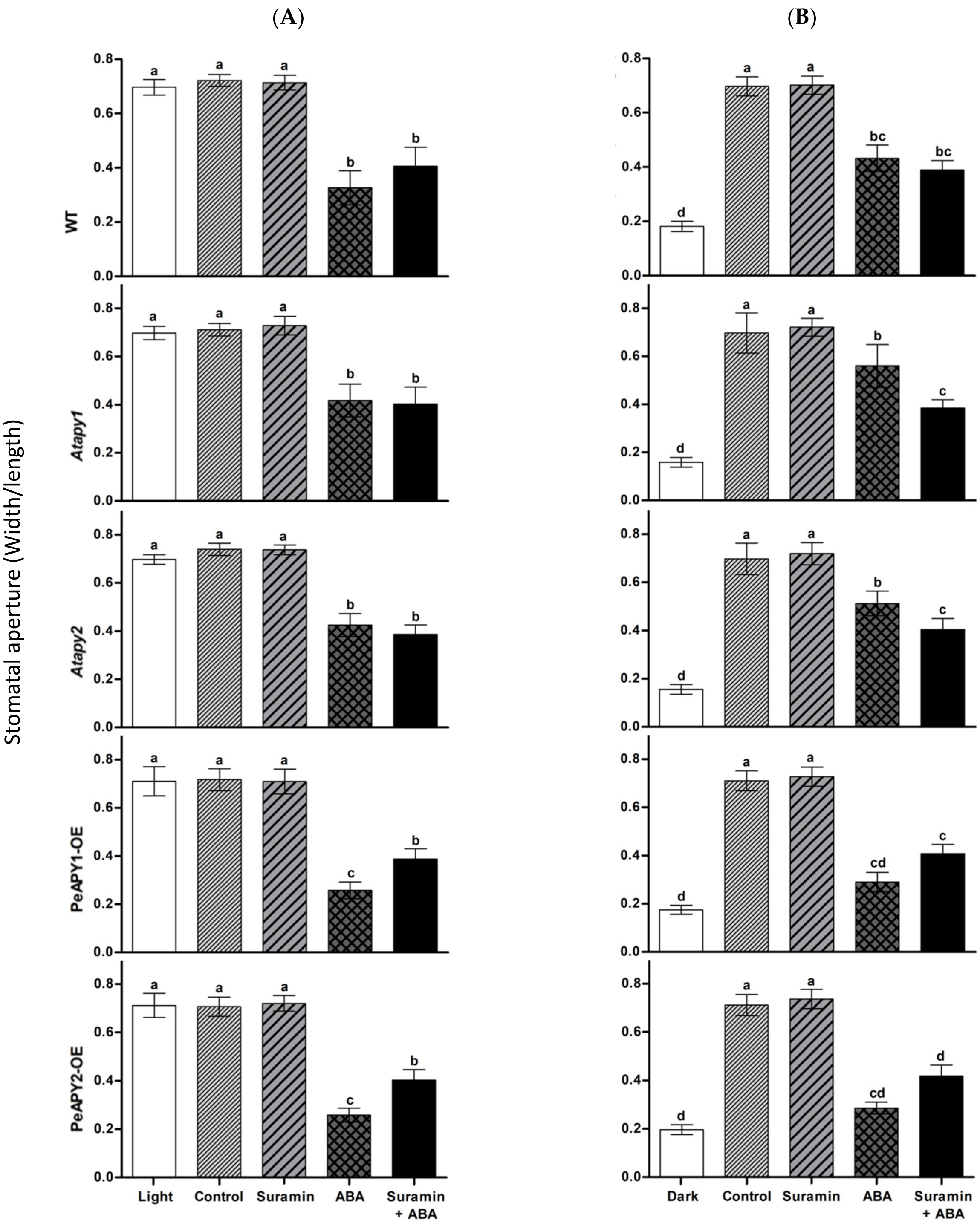

Figure S3. Effect of purinergic receptor antagonist (suramin) on ABA-regulated stomatal aperture in wild type (WT), Arabidopsis loss-of-function mutants for *APY1* and *APY2* (*Atapy1* and *Atapy2*), and transgenic lines of *PeAPY1* and *PeAPY2* (PeAPY1-OE and PeAPY2-OE). Leaves from three-week-old seedling were incubated in MES-Tris buffer containing 50 mM KCl and 10 mM MES-Tris (pH 6.15) for 2 h in light (150 μmol m^{-2} s^{-1}) in (**A**) or in darkness (**B**). Thereafter leaves were exposed to 100 μM suramin for 2 h in cool light (150 μmol m^{-2} s^{-1}) in the presence and absence of ABA (10 μM). Controls were treated without the addition of inhibitor or ABA. Stomatal aperture was measured in continuously illuminated leaves (**A**) and dark-adapted plants transferred to light (**B**), respectively. Each column is the mean of three independent experiments, and error bars represent SE. Columns labeled with different letters, a, b, c, d, showed significant difference at $P < 0.05$ between treatments and genotypes under conditions of continuous light (**A**) or dark-adapted leaves transferred to light (B).
