# Supplementary material for: Populus euphratica Apyrases Increase Drought Tolerance by Modulating Stomatal Aperture in Arabidopsis
Source: Int J Mol Sci. 2021 Sep 13;22(18):9892. doi: 10.3390/ijms22189892 (PMC8468604; doi:10.3390/ijms22189892)
Supplement: Supplementary file 1 [file ijms-22-09892-s001.zip › Figure S4_corrected.pdf]

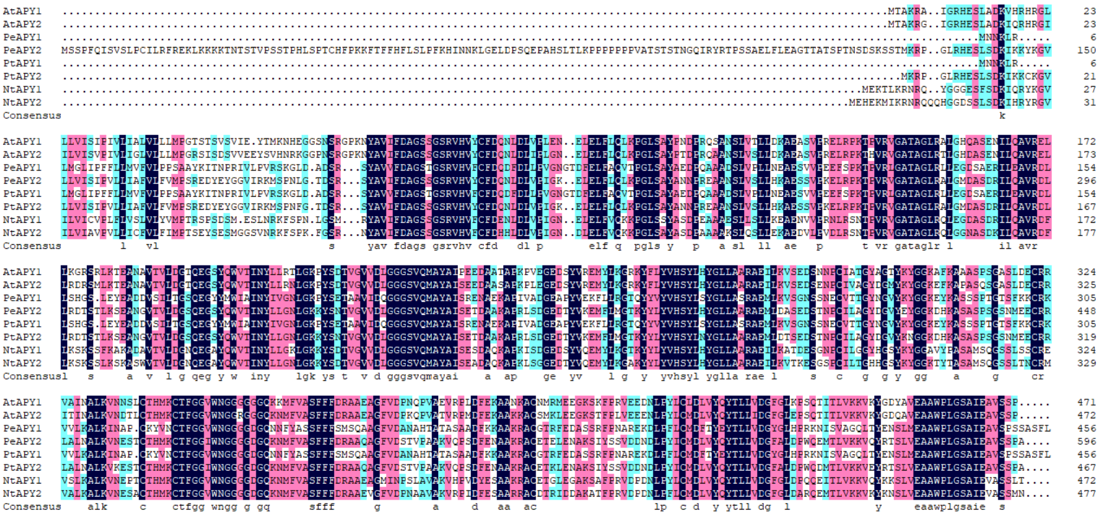

Figure S4. Multiple sequence alignment of PeAPY1 and PeAPY2 (apyrase from *Populus euphratica*) with other apyrases from different species. The APY sequences are from *P. euphratica* (PeAPY1, PeAPY2), *Populus tricocarpa* (PtAPY1, PtAPY2), *Arabidopsis thaliana* (AtAPY1, AtAPY2) and *Nicotiana tobacum* (NtAPY1, NtAPY2). Full length amino sequences of APYs were obtained by similarity searching on NCBI website (http://www.ncbi.nlm.nih.gov/). The accession numbers of these sequences are provided in Supplemental Table S1. The multiple sequence alignment of amino sequence was performed by ClustalW. Shadings in black, red, blue, indicate identical and conserved amino acid residues.
