# Supplementary material for: Populus euphratica Apyrases Increase Drought Tolerance by Modulating Stomatal Aperture in Arabidopsis
Source: Int J Mol Sci. 2021 Sep 13;22(18):9892. doi: 10.3390/ijms22189892 (PMC8468604; doi:10.3390/ijms22189892)
Supplement: Supplementary file 1 [file ijms-22-09892-s001.zip › Figure S5_corrected.pdf]

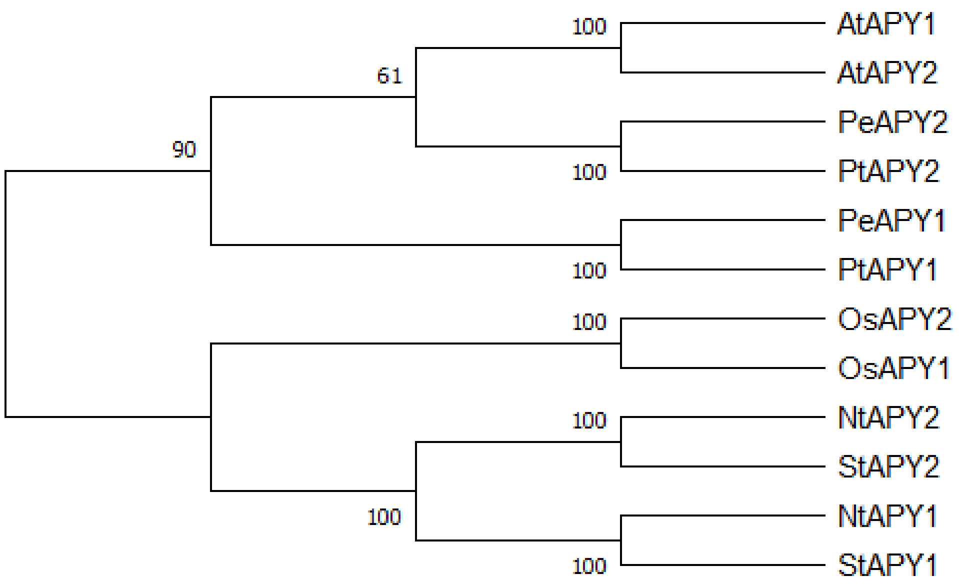

Figure S5. Phylogenetic relationships among PeAPY1, PeAPY2 and other representative APY proteins from different plant species. Apyrases in different species are indicated as follows: PeAPY1, PeAPY2, *Populus euphratica*; StAPY1, StAPY2, *Solanum tuberosum*; AtAPY1 and AtAPY2, *Arabidopsis thaliana*; PtAPY1, PtAPY2, *Populus tricocarpa*; NtAPY1, NtAPY2, *Nicotiana tobacum*; OsAPY1, OsAPY2, *Oryza sativa*. The phylogenetic trees were constructed via Neighbor-joining program of MEGA 5.2 software. Bootstrap values of 1000 replicates are shown at the nodes. The accession numbers of the APY sequences are provided in Supplementary Table S1.
