# Supplementary material for: Populus euphratica Apyrases Increase Drought Tolerance by Modulating Stomatal Aperture in Arabidopsis
Source: Int J Mol Sci. 2021 Sep 13;22(18):9892. doi: 10.3390/ijms22189892 (PMC8468604; doi:10.3390/ijms22189892)
Supplement: Supplementary file 1 [file ijms-22-09892-s001.zip › Table S1_corrected.pdf]

Table S1. Accession numbers of apyrase orthologs used in multiple sequence alignment and phylogenetic analysis.

Sequence name	Accession number	Sequence name	Accession number
AtAPY1	NP_187058.1	AtAPY2	NP_197329.5
NtAPY1	XP_016476679.1	NtAPY2	XP_016463837.1
OsAPY1	XP_015632681.1	OsAPY2	XP_015647007.1
PeAPY1	XM_011001170.1	PeAPY2	XM_011036124.1
PtAPY1	XP_002325398.1	PtAPY2	XP_002319138.3
StAPY1	XP_006364259.1	StAPY2	XP_006342448.1
