# Supplementary material for: Populus euphratica Apyrases Increase Drought Tolerance by Modulating Stomatal Aperture in Arabidopsis
Source: Int J Mol Sci. 2021 Sep 13;22(18):9892. doi: 10.3390/ijms22189892 (PMC8468604; doi:10.3390/ijms22189892)
Supplement: Supplementary file 1 [file ijms-22-09892-s001.zip › Table S2_corrected.pdf]

Table S2. Primers used for quantitative real-time PCR

Gene	Forward primer (5´-3´)	Reverse primer (5´-3´)
RBOHF	CCAATGTCCTGCGGTTTC	TTGTTGTTTCGTCGGCTC
RBOHD	GATGGGAGACAGCAGGAT	TTTCGACAAACAGGCAAC
AtACTIN2	GGTAACATTGTGCTCAGTGGTGG	AACGACCTTAATCTTCATGCTGC
